# Reveal the Regulation Patterns of Prognosis-Related miRNAs and lncRNAs Across Solid Tumors in the Cancer Genome Atlas

**DOI:** 10.3389/fcell.2020.00368

**Published:** 2020-05-25

**Authors:** Zuojing Yin, Qiming Wang, Xinmiao Yan, Lu Zhang, Kailin Tang, Zhiwei Cao, Tianyi Qiu

**Affiliations:** ^1^Department of Gastroenterology, Shanghai Tenth People’s Hospital, School of Life Sciences and Technology, Tongji University, Shanghai, China; ^2^Shanghai Public Health Clinical Center, Fudan University, Shanghai, China

**Keywords:** pan-cancer prognosis analysis, solid tumor, miRNAs, lncRNAs, regulation pattern

## Abstract

**Background:**

The dysregulation of non-coding RNAs (ncRNAs) such as miRNAs and lncRNAs are associated with the pathogenesis and progression in multiple cancers including solid tumors. Comprehensive investigations of prognosis-related ncRNA markers could promote the development of therapeutic strategies for solid tumors, but rarely reported.

**Methods:**

By taking advantage of The Cancer Genome Atlas (TCGA), pan-cancer prognosis analysis (PCPA) models were firstly constructed based on miRNA and lncRNA expression profiles of 8,450 samples in 19 solid tumors. Further, the co-occurrence and exclusivity among ncRNA markers were systematically analyzed for different cancers.

**Results:**

In identified ncRNA makers, 71% of the miRNA markers were shared in multiple cancers, whereas 96% of the lncRNA markers were cancer-specific. Moreover, to analyze the regulation patterns of prognosis-related ncRNAs at the pan-cancer level, miRNA markers were further annotated into eight carcinogenic pathways. Results represented that approximately 86% of these miRNA markers could regulate the PI3K-Akt signaling pathway, while only 48% for the Notch signaling pathway. Finally, among 126 common genes that participated in eight carcinogenic pathways, BCL2, CSNK2A1, EGFR, PDGFRA, and VEGFA were proposed as potential drug targets for multiple cancers.

**Conclusion:**

The prognosis analysis and regulation characteristics of ncRNAs presented in this study may help to facilitate the discovery of anti-cancer drugs for multiple solid tumors.

## Introduction

Despite a variety of treatments for patients with solid tumor including radiotherapy (Verduin et al., 2017), phototherapy (Song et al., 2017), chemotherapy (Grossman et al., 2015), chemophototherapy (Luo et al., 2017), surgical resection (Heaton and Davidoff, 2016) and immunotherapy (Chen et al., 2017), etc., it has been recognized that patients often suffer from incomplete killing of tumor cells (Luo et al., 2017), drug resistance (Lorz et al., 2015) and poor prognosis (Wang et al., 2017). Thus, analysis of the prognostic characteristics is critical for detecting appropriate therapeutic applications and improving the OS of cancer patients. Moreover, identification of novel and predictive prognostic biomarkers for both intra- and inter- cancer types is warranted in the management and classification of cancer cases during or after the therapy, as well as prognosis prediction of patients at the earlier stages (Bahrami et al., 2018). Also, PCPA would guide the development of individual and universal therapeutic approaches for multiple cancer types (Gaforio et al., 2003; Su et al., 2014).

Recently, with the development of sequencing technology, the “dark matter” of genomes which called “ncRNAs” has been excavated and proved to play an essential role in life regulation processes (Mitra et al., 2012). Based on the transcript size, ncRNAs can be further divided into two subclasses including (1) small ncRNA (20–200 nt), such as miRNA, and (2) long ncRNAs (>200 nt), such as lncRNAs (Gupta et al., 2010; Tzadok et al., 2013; Liu M. X. et al., 2014). Further research implicated that ncRNAs altered in various malignancies (Costa, 2005; Calin and Croce, 2006), which is crucial in the modulation of tumor behaviors (Mitra et al., 2012). Also, growing evidence indicated that ncRNAs influenced the onset, progression, and outcomes of cancer (Liu J. H. et al., 2014), which could be used as diagnosis and prognosis markers in the primary malignancy to determine long-term prognosis (Mitra et al., 2012). Several shreds of evidence such as HOTAIR (Gupta et al., 2010; Kim et al., 2013; Nakagawa et al., 2013), MEG3 (Lu et al., 2013), and LOC285194 (Qi et al., 2013) were proved to be closely associated with survival and reported as prognostic indicators for specific cancer types. Thus, the identification of robust and reproducible ncRNA markers at the pan-cancer level would not only help to reveal the complexity and heterogeneity of cancer from regulation perspectives, but also improve the cancer-specific treatment and personalized medication. However, studies dedicated to evaluate and compare the prognostic difference at the pan-cancer level are rarely reported.

By taking advantage of the Pan-Cancer project of TCGA (Tomczak et al., 2015), it is possible to comprehensively analyze the prognosis at the pan-cancer level through high-throughput ncRNA expression data and clinical information. In this study, the PCPA models for 19 solid tumors involving 8,450 patient samples were firstly constructed by integrating the expression of miRNAs and lncRNAs. The characteristics of prognosis-related ncRNAs indicated that miRNAs prefer to regulate multiple cancer types, while lncRNAs tend to be cancer-specific. Furthermore, the regulation patterns of miRNA markers were depicted in eight canonical pathways. Moreover, based on the MOS process, both specific genes for different cancer categories and common genes for 19 solid tumors which could be regulated by prognosis-alternative miRNA markers were proposed as potential drug targets. In general, the results illustrated here could not only reveal the regulation pattern of cancer-related ncRNAs on canonical signaling pathways, but also guide the potential therapeutic applications for multiple solid tumors.

## Materials and Methods

### Data Source

The miRNA expression profiles of 19 solid tumors from the IlluminaHiseq platform, as well as the clinical information of corresponding tumors were obtained from the TCGA module of Xena Public Data Hubs in the UCSC Xena platform (Goldman et al., 2017). For each sample, all isoform expressions for the same miRNA mature strand were added together and transformed with logarithm. The original number of patient samples and miRNAs in each cancer type were showed in Supplementary Table 1. Besides, the lncRNA expression profiles of the above 19 solid tumors were obtained from The Atlas of Non-coding RNAs in Cancer (TANRIC) database 1.0.7 (Li et al., 2015) based on TCGA (Tomczak et al., 2015), which quantified the expression levels of lncRNAs with RPKM based on the BAM files. And, the numbers of original samples and lncRNAs in each cancer type were also shown in Supplementary Table 1. Both the expression profiles of miRNAs and lncRNAs were derived from RNA-seq data.

Here, we pre-processed the ncRNA expression profiles by filter out those not available (NA) values. Typically, the ncRNAs markered with “NA” in at least one sample, as well as those samples marked with “NA” in at least one ncRNA were filtered out in our study. Moreover, the miRNA accessions in the filtered miRNA expression profiles were further converted into miRNA IDs according to the miRbase database (Kozomara et al., 2019), and the expression values of the same miRNA ID were integrated by the average values in the same sample. Then, the expression profiles of lncRNAs were intersected with the clinical samples contains OS. Samples with OS markered with “NA” and identified as normal tissue by the TCGA sample nomenclature were filtered out. The numbers of samples and ncRNAs after pre-processing were displayed in Supplementary Table 2. Thus, the total number of 8,450 samples with survival information were selected as research objects. Among them, 6,061 samples contain lncRNA expression profiles and 7,203 samples contain miRNA expression profiles. For patient samples, the median OS in each cancer type was considered as the prognosis classification indicator.

The genes which were regulated by corresponding prognosis-related miRNA markers were annotated through three high-quality experimentally validated miRNA-target interaction databases, including miRTarbase 7.0 (Chou et al., 2018), miRecords 2013 (Xiao et al., 2009), and TargetScan 3.1 (Riffo-Campos et al., 2016). Genes of canonical signaling pathways were downloaded from the KEGG database (Kanehisa, 2002). Monotherapy and combinational therapy for multiple solid tumors were extracted from NCBI PubMed, DCDB 2.0 (Liu Y. et al., 2014) and DrugCombDB (Liu et al., 2020) database. The corresponding drug targets were derived from drugBank 5.1.4 (Wishart et al., 2018) and TTD 2019 (Li et al., 2018) database.

### Detecting Differential Expressed ncRNAs

To identify prognosis-related ncRNAs, essential ncRNAs of each solid tumor were selected by taking the median OS as a classification indicator. For each solid tumor, patient samples were divided into high-OS and low-OS groups according to their corresponding median OS evaluated with the original clinical matrix (Supplementary Table 3). Specifically, patients of each cancer type with OS over than its median value were regarded as high-OS (positive) group, otherwise, low-OS (negative) group. Further, two-tailed *t*-tests were used to evaluate the differential expressed (DE) ncRNAs between positive and negative samples. For the lncRNA expression profiles, the DE lncRNAs were filtered with conditions of *P* < 0.01 and absolute fold change value | FC| > 2, and the DE miRNAs were filtered with *P* < 0.05. The DE ncRNAs in each cancer type was the combination of the DE miRNAs and lncRNAs, and the samples were the intersection of samples in miRNAs and lncRNAs expression profiles of the corresponding cancer. Note that, the number of training samples is quite small in merged ncRNAs for KICH (45 samples) and LUSC (44 samples), which may lead to the overfitting of PCPA modeling. Thus, factor analysis was performed to reduce the dimensions of the combined ncRNAs in KICH and LUSC by the *psych* package 1.9.12.31 of R software (Lorenzo-Seva and Van Ginkel, 2016b), and the top 10 lncRNAs for each factor were selected according to the weight matrix to identify the prognosis-related ncRNAs. The expression profiles of DE miRNAs, lncRNAs, as well as combined ncRNAs of each cancer, were used for subsequent modeling.

### Construction of Pan-Cancer Prognosis Analysis (PCPA) Model

Training and testing datasets of each solid tumor were obtained through the spatial subset sampling method to generate PCPA models. Typically, the first sample A was randomly selected as the seed, and the second sample B with the farthest spatial distance from sample A was selected. Next, the third sample with the farthest average distance toward both samples A and B was extracted. Then, sampling was repeated until two-thirds of the positive and negative samples were screened as the training set, and the rest samples were defined as the testing set. Both single-omic and two-omic ncRNA datasets of 19 solid tumors were used to construct the PCPA model. Here, four machine learning models including NN, NB, LR, and SVMs were implemented by using the python 2.7.9 *sklearn* package 0.3.6 (Lorenzo-Seva and Van Ginkel, 2016a) to generate the PCPA model based on labels divided from the median OS of corresponding patient samples.

### Survival Analysis

Survival analysis (Wang et al., 2019) was performed based on the classification results of different PCPA models. KM survival curves of different samples were evaluated by using the R *survival* 3.1-11 and *survminer* 0.4.6 package (Modhukur et al., 2018). In addition, the log-rank test (Rantala et al., 2019) was employed to test the difference between the two compared sample groups.

### Construction of Refined Gene-Specific Pathway

Genes regulated by corresponding prognosis-related miRNA markers were obtained from miRNA-target interaction databases, including miRTarbase 7.0 (Chou et al., 2018), miRecords 2013 (Xiao et al., 2009), and TargetScan 3.1 (Riffo-Campos et al., 2016). Genes that were regulated by prognosis-related lncRNA markers were converted from gene ENSEMBL to gene SYMBOL by the *org.Hs.eg.db* 3.7.0 (Prummer, 2019) and the *clusterProfiler* 3.14.3 (Yu et al., 2012) package in R software.

Further, eight canonical signaling pathways with frequent genetic alterations in cancers regulated (Sanchez-Vega et al., 2018) by the above detected prognosis-related ncRNAs were evaluated. After annotation to the KEGG database (Kanehisa, 2002), the gene list of these eight pathways was obtained and the original ENTREZ ID of the gene in each pathway was converted to SYMBOL by the *org.Hs.eg.db* 3.7.0 (Prummer, 2019) and *clusterProfiler* 3.14.3 (Yu et al., 2012) package in R software. For each pathway, the intersected genes with the remaining seven pathways were removed to construct the refined gene-specific pathway.

### Establish the Marker-Oriented Simulation (MOS) Process for Patient Samples

To detect potential drug targets for novel therapeutic strategies, the MOS process was provided. All TCGA samples in our testing set were pre-clustered as high-OS and low-OS groups according to the classifier indicator of median OS. Since currently approved anti-cancer drugs were mostly inhibitors that could down-regulate the expression of cancer-related genes or proteins (Wishart et al., 2018), and miRNAs have been proved to generally negatively regulate gene expressions (Di Leva and Croce, 2013), miRNA markers that were highly expressed in high-OS groups might provide similar inhibition ability as anti-cancer drugs. Thus, for each cancer category, prognosis-related miRNA markers in low-OS groups with lower expression levels were alternately adjusted to high levels as those in high-OS groups. Those miRNAs that could increase the prediction possibility of high-OS were detected as prognosis-alternative miRNAs.

## Results

### Diversity of ncRNA Datasets for Solid Tumors

**FIGURE 1:**
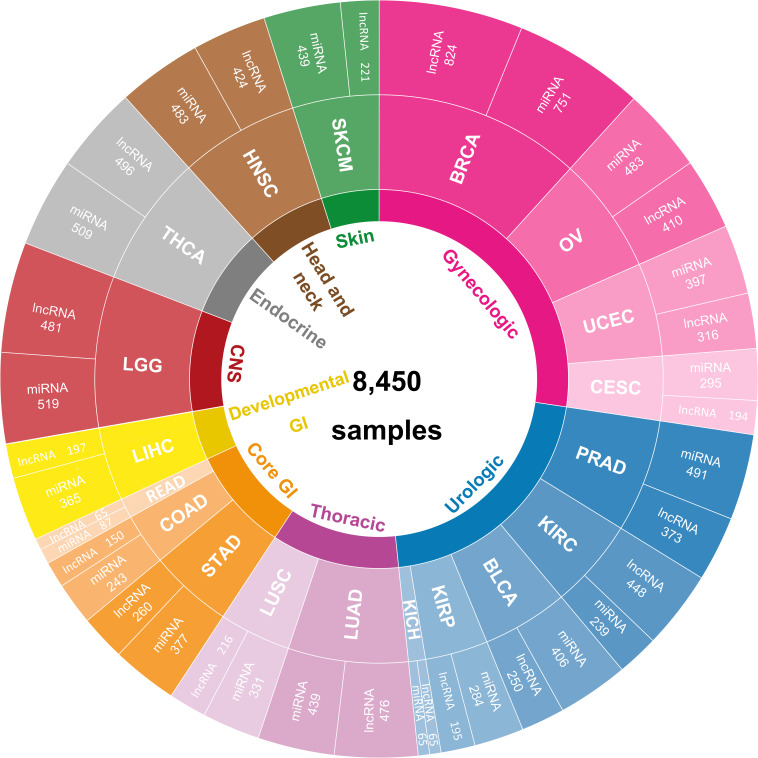
The pan-cancer datasets collated in this study. Data distribution of corresponding ncRNA samples for 19 cancer types in nine cancer categories.

To analysis the pan-cancer prognosis through ncRNAs, expression profiles of miRNA and lncRNA with corresponding clinical information for patient samples were collected here. After quality control, 19 solid tumors with available miRNA and lncRNA expression profiles derived from RNA-seq have remained as our datasets. According to the previous report (Hoadley et al., 2018), these 19 solid tumors could be clustered into nine categories, which includes urologic cancer (bladder cancer [BLCA], kidney chromophobe [KICH], kidney clear cell carcinoma [KIRC], kidney papillary cell carcinoma [KIRP], prostate cancer [PRAD]), gynecologic cancer (breast cancer [BRCA], cervical cancer [CESC], ovarian cancer [OV], endometrioid cancer [UCEC]), core gastrointestinal (GI) and development GI cancer (colon cancer [COAD], rectal cancer [READ], stomach cancer [STAD], liver cancer [LIHC]), Thoracic cancer (lung adenocarcinoma [LUAD], lung squamous cell carcinoma [LUSC]), central nervous system (CNS) cancer (lower grade glioma [LGG]), Head and neck cancer (head and neck cancer [HNSC]), Endocrine cancer (thyroid cancer [THCA]), and melanocytic cancers of the skin (melanoma [SKCM]). Detailed information can be found in Supplementary Table 4. The most abundant category of urologic cancer contains five cancer subtypes, and followed by gynecologic, core GI, thoracic, CNS, head and neck, endocrine, developmental GI and melanocytic cancers of the skin. In general, 8,450 TCGA samples with miRNA and lncRNA expression profiles were collected here, sample numbers for each cancer type were illustrated in Figure 1.

### Prognosis-Related ncRNA Markers for Solid Tumors

To identify prognosis-related ncRNAs for 19 solid tumors, DE ncRNAs which associated with prognosis were initially identified for different cancer types by setting appropriate filters and factor analysis (see section “Materials and Methods”). After removing redundancy, prognosis-related ncRNAs for different cancer types were identified and listed in Supplementary Table 5. Among them, LGG contains 168 prognosis-related ncRNAs which including 73 miRNAs and 95 lncRNAs, while UCEC only contains 24 miRNAs and 1 lncRNA, indicated the diversity of 19 solid tumors.

**FIGURE 2:**
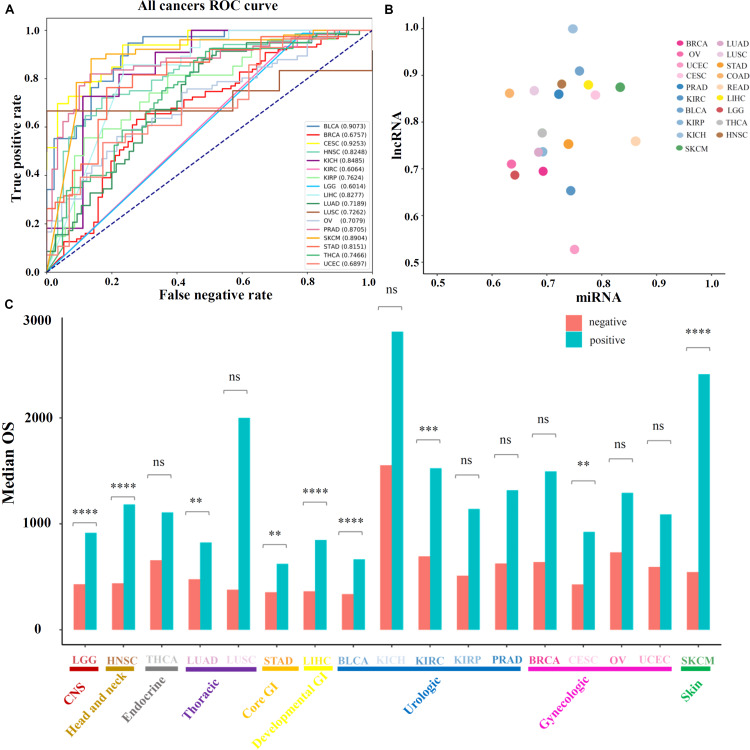
Prediction performance of prognosis-related ncRNA markers. **(A)** The ROC curves of 19 solid tumors with prognosis-related ncRNA markers. **(B)** The AUC values of 19 cancer types, in which the X-axis represents the AUC values based on prognosis-related miRNAs and Y-axis represents the AUC values based on prognosis-related lncRNAs. **(C)** The median OS values and statistical significance of high-OS and low-OS samples for all 19 cancer types. Here, the *P*-values of the log-rank test in survival analysis were provided.

Further, the performance of the above prognosis-related ncRNAs was evaluated for distinguishing high-OS and low-OS samples through PCPA models which were constructed for 19 solid tumors (see section “Materials and Methods”), the numbers of total samples, training samples and selected features for PCPA modeling were showed in Supplementary Table 6 as been illustrated in Supplementary Table 6. The numbers of the total samples, training samples and selected features for PCPA modeling were shown in Supplementary Table 6. Then, ROC curves and the AUC value were introduced for model validation. Through overall evaluation, the NB model revealed the generally good prediction performance compared with other machine learning approaches, and was chosen for PCPA modeling and further prognosis analysis. The ROC curves of 19 solid tumors represented diverse classification performance based on identified prognosis-related ncRNAs with AUC value from 0.60 to 0.93 (Figure 2A), which could be further increased from 0.67 to 0.93 by selecting the most suitable machine learning approaches for each cancer type (Suppelmentary Table 7). Then, the AUC values of 19 solid tumors based on lncRNAs and miRNAs were displayed in Figure 2B and Supplementary Table 7, in which most of the prediction models could achieve an AUC value of over 0.70. Besides classifications, the prognosis-related ncRNAs were evaluated through survival analysis based on the prediction of PCPA models, and the barplot of median OS in predicted positive and negative samples were illustrated in Figure 2C, with the statistical significance evaluated by log-rank test in survival analysis. Also, the corresponding log-rank *P*-values of survival analysis in 19 solid tumors were displayed in Supplementary Table 8. The results of model prediction and survival analysis showed that ncRNAs identified here could distinguish the high-OS and low-OS samples, which were considered as prognosis-related markers for further analysis.

### Cancer-Specific and Pan-Cancer Regulation Patterns of ncRNA Markers

**FIGURE 3:**
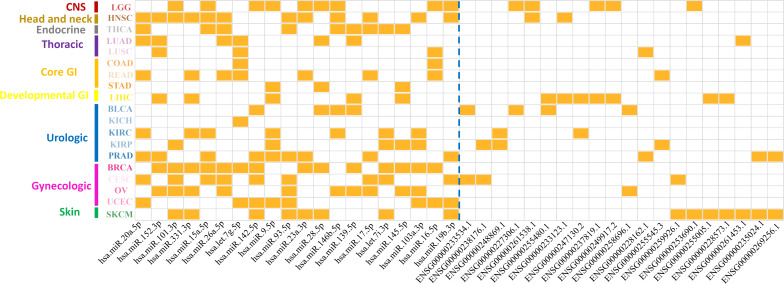
Distribution patterns of top-ranking ncRNA markers on associated cancer types. The x-axis represents the top 20 miRNA markers and lncRNA markers divided by a blue dotted line. Y-axis represents 19 cancer types in nine cancer categories. Each grid marked with orange colors represents the ncRNA markers were associated with the corresponding cancers.

After removing redundancy between different cancer types, a total number of 305 miRNA markers and 599 lncRNA markers were determined as prognosis-related markers in 19 solid tumors (Supplementary Table 9). It is noted that the regulation patterns of miRNA and lncRNA markers were different from each other. In fact, 216 out of 305 (∼71%) miRNA markers shared in multiple cancer types, while only 23 out of 599 (∼4%) of the lncRNA markers shared in more than one cancer type. Furthermore, the top 20 markers for both miRNA and lncRNA, which were ranked by the number of corresponding cancer types, were illustrated in Figure 3. Interestingly, miRNA markers could not only enriched in different cancer types for one category, but also in multiple cancer categories. For example, hsa.miR.152.3p and has.let.7g.5p were associated with the prognosis of all thoracic cancers involved in our study. Also, it can be found that hsa.miR.20a.5p was identified in six cancer categories including head and neck, endocrine, thoracic, GI, urologic, and gynecologic. Thus, miRNAs might prefer to affect the prognosis at the pan-cancer levels.

**FIGURE 4:**
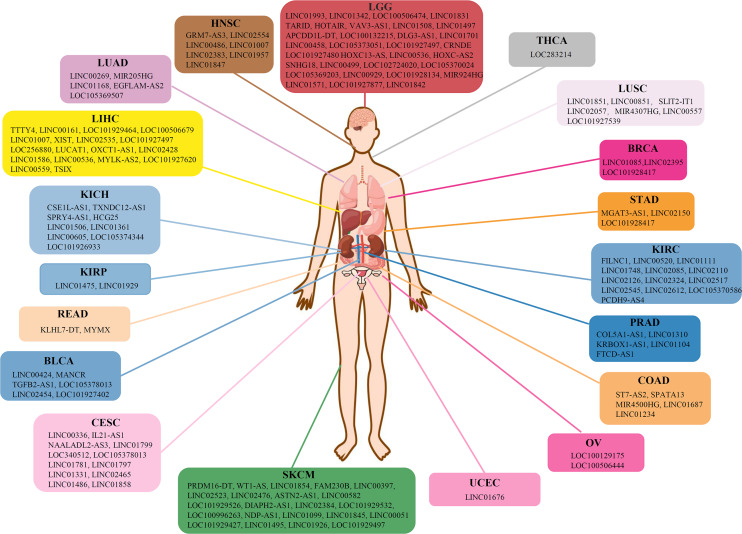
Gene symbols of corresponding lncRNA markers in 19 solid tumors.

On the contrary, lncRNAs reflected a tendency to influence the cancer prognosis specifically, in which only ∼4% of the lncRNA markers were observed in more than one cancer type (Figure 3). To further reveal the regulation patterns, 599 prognosis-related lncRNA markers in 19 solid tumors were converted into 150 corresponding gene symbols by R package *org.Hs.eg.db* 3.7.0 and *clusterProfiler* 3.14.3 (see section “Materials and Methods”), and have been illustrated in Figure 4. Among them, several markers that detected here have been proved to be associated with the occurrence, progression, and prognosis of corresponding cancers. Such as HOTAIR was reported to significantly associated with the invasiveness of gliomas (Zhao et al., 2019).

**FIGURE 5:**
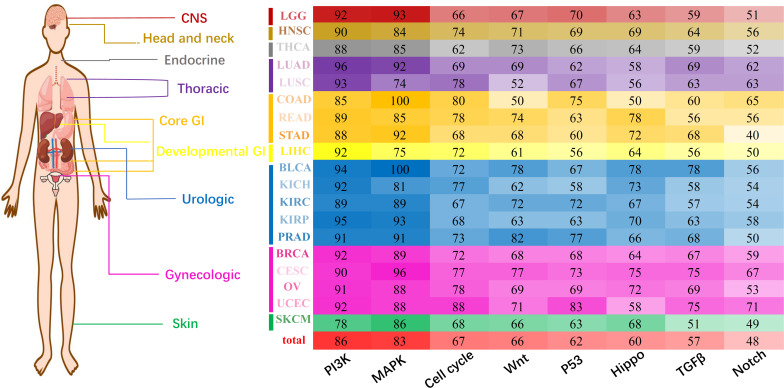
Pan-cancer regulation of miRNA markers in canonical pathways. The X-axis represents eight different essential canonical pathways including PI3K, MAPK, Cell cycle, Wnt, P53, Hippo, TGFβ, and Notch. Y-axis represents 19 cancer types and corresponding cancer categories. Different numbers and colors represent the regulation percentage of corresponding miRNA markers.

Considering that miRNA tended to interfere with cancer prognosis at the pan-cancer level, we tried to reveal the regulation characteristics of miRNA markers through oncologic signaling pathways. According to the database of miRTarBase 7.0 (Chou et al., 2018), miRecords 2013 (Xiao et al., 2009), and TargetScan 3.1 (Riffo-Campos et al., 2016), 15714 genes were found to be regulated by 305 miRNA markers in 19 solid tumors (see section “Materials and Methods”). Regulation patterns of miRNA markers were analyzed through eight essential canonical signaling pathways which involving: (1) PI-3-Kinase (PI3K) signaling, (2) MAP-Kinase (MAPK) signaling, (3) cell cycle, (4) Wnt signaling, (5) P53 signaling, (6) Hippo signaling, (7) TGFβ signaling, and (8) Notch signaling pathways (Sanchez-Vega et al., 2018). To generate the refined gene-specific pathway, common genes shared in other pathways were removed and the remained genes consisted of refined gene-specific pathways (Supplementary Table 10). Finally, for each cancer type, the regulation characteristics of miRNA markers in canonical signaling pathways were displayed in Figure 5.

Generally, all eight canonical pathways were enriched with genes regulated by prognosis-related miRNA markers. In fact, approximately 86% of the miRNA markers in 19 solid tumors were discovered to regulate gene targets in PI3K signaling pathways, and followed by the MAPK signaling pathway which involving 83% of the miRNA markers. Besides the above, the Cell cycle, Wnt, P53, Hippo and TGFβ signaling pathways were regulated by over 50% of the miRNA markers, and only 48% of the miRNA markers were detected to regulate the corresponding genes in the Notch signaling pathway. For most of the solid tumors analyzed here, the regulation characteristic of miRNA markers was similar to the general profiles, which were mainly participated in the regulation of PI3K and MAPK signaling pathway, and followed by the other six pathways.

### Common Genes and Potential Therapeutic Applications

**FIGURE 6:**
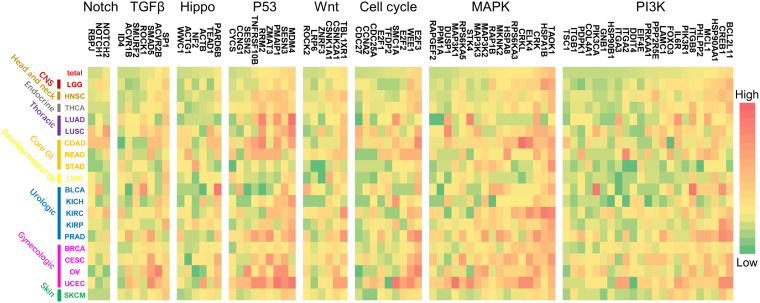
The regulation patterns of genes in canonical pathways for multiple cancer types. For each pathway, the genes that were regulated by over 7% of the miRNA markers were illustrated. For each cancer type, different color represents the regulation percentage of corresponding miRNA markers.

Furthermore, the regulation patterns of genes in the above refined gene-specific pathway were analyzed for different cancer types. In each refined gene-specific pathway, genes that were regulated by over 7% of total miRNA markers were illustrated in Figure 6, and the results of regulation over 5% can be found in Supplementary Figure 1. For the PI3K signaling pathway, 23 genes were detected, which was partially consistent with previous studies. Such as BCL2L11 and MAP3K2 were considerably regulated by miRNA markers in UCEC and PRAD of our study (Figure 6), which were consistent with the previous studies (Fulford et al., 2016; Fialkova et al., 2017).

Genes in the canonical pathways, which were regulated by miRNA markers, hold the potential to guide clinical trials for targeted therapies in multiple cancer types. Thus, common genes among different cancer types or major cancer categories might inform the choice of therapeutic targets. To detect potential drug targets for multiple cancers, MOS modeling was performed to detect prognosis-alternative miRNA markers (see section “Materials and Methods”). Since anti-cancer drugs tend to intervene with genes in cancer-associated pathways, potential drug targets were examined from genes that participated in cancer-related pathways. Here, four major cancer categories including urologic, gynecologic, GI and thoracic cancer were applied for MOS analysis to prompt potential therapeutic drug targets. The prognosis-alternative miRNA markers for each cancer category through the MOS process were shown in Supplementary Table 11.

In urologic cancer, 139 miRNA markers regulating 243 common genes were derived. Through the process of MOS, common genes that could be regulated by prognosis-alternative miRNA markers, such as BCL2, EGFR, KIT, PDGFRA, and VEGFA were currently validated as drug targets. For example, KIT and PDGFRA is the target for Sorafenib (Wishart et al., 2018; Abdelgalil et al., 2019) and Sunitinib (Poveda et al., 2017; Wishart et al., 2018), respectively. For gynecologic cancer, 387 common genes were regulated by 148 miRNA markers. Detected potential drug targets, such as BCL2, EGFR, PDGFRA, and VEGFA were proved to be drug targets for previously approved drugs. Such as EGFR is the target for trastuzumab (Chen et al., 2002; Wishart et al., 2018). In GI cancer, 254 common genes were examined in four cancer subtypes and regulated by 92 miRNAs. Among them, genes such as EGFR, PDGFRA, VEGFA, PDPK1, and MCL1 could be regulated by prognosis-alternative miRNAs. As reported previously, Imatinib could target MCL1 (Wishart et al., 2018; Wang et al., 2020). Similarly, in thoracic cancer, 363 common genes were regulated by 50 miRNA markers. Prognosis-alternative miRNA regulated genes such as BCL2, EGFR, IGF1R, VEGFA, and MET were validated as drug targets. For example, Crizotinib could target MET (Heigener and Reck, 2018; Wishart et al., 2018). Detailed information on common genes that were regulated by miRNA markers and annotated in eight canonical pathways for the above four major categories was summarized in Supplementary Table 12, and common genes regulated by prognosis-alternative miRNAs through MOS process proposed potential drug targets (Supplementary Table 13). Finally, 126 common genes regulated by miRNA markers were observed in 19 solid tumors. Among them, genes such as BCL2, CSNK2A1, EGFR, PDGFRA, VEGFA, etc., which have been proved to be targets of anti-cancer drugs, were proposed to provide general administrations for pan-cancers.

## Discussion

Non-coding RNAs, which can not be directly translated into proteins, have been proved to be involved in the large-scale regulation of many protein-coding genes and contribute various complex diseases, including cancers (Farh et al., 2005; Lim et al., 2005). It has been observed that many ncRNAs showed abnormal expression patterns in cancerous tissues such as miRNAs and lncRNAs (Christov et al., 2008; Shahrouki and Larsson, 2012). Revealing the regulation characteristics of ncRNAs at the pan-cancer level is essential for the diagnosis, monitoring, treatment and therapeutic development for cancer patients. In this study, the pan-cancer prognosis of 19 solid tumors was analyzed by integrating miRNAs and lncRNAs in TCGA patients. By deriving prognosis-related ncRNA markers, PCPA models were established for each cancer type. Since the NB model could generally achieve good predictive effects in most of the solid tumors, in this study, NB was selected as the predictive method in the PCPA models. Prediction results showed that even though most of the PCPA models could significantly distinguish high-OS and low-OS samples, the prediction performances remained variant for different cancers. For example, the AUC value of SKCM based on miRNA markers could reach to 0.83, while COAD could only achieve to 0.63. Further analysis showed that 59 miRNA markers were detected for SKCM and only 20 for COAD. On the contrary, the AUC value of LGG which based on 95 lncRNA markers could only reach 0.69, while BLCA could achieve to 0.91 through 35 lncRNA markers. Thus, the regulation patterns for ncRNAs might present diversities among different solid tumors analyzed here.

Moreover, the co-occurrence and exclusivity of prognosis-associated miRNAs and lncRNAs among 19 cancers were analyzed to reveal their distribution patterns. It is noted that almost 71% of the miRNA markers shared in multiple cancer categories or different cancer subtypes within one category, indicating the preference of miRNAs to regulating prognosis at the pan-cancer level. On the other hand, the regulation of lncRNA markers reflects to be cancer-specific, in which approximately 96% of them merely appeared to have participated in the prognosis of only one cancer type. Since these prognosis-related miRNAs could regulate the expression of multiple genes involved in essential canonical cancer pathways at the pan-cancer levels, their regulation patterns on cancer pathways or pathway genes could help illustrate the influence of miRNAs in cancer prognosis. Regulation results showed that miRNA markers were preferred to intervene PI3K and MAPK signaling pathways, while less in Notch signaling pathways (Figure 5). The top 7% genes of cancer-related pathways regulated by miRNA markers were proved to be involved in the development of the corresponding cancers. For example, BCL2L11 regulated by miRNA markers in UCEC (Figure 6), was proved to be differentially expressed between endometrial canceration and normal menstrual cycles (*P* < 0.0001) (Fialkova et al., 2017). The MAP3K2 gene in the MAPK pathway, which was regulated by the miRNA markers in PRAD, was previously reported to be associated with the growth and metastasis of prostate tumors (Fulford et al., 2016). Also, target genes regulated by these miRNA markers and annotated in canonical pathways would hold the potential to serve as drug targets. Since current anti-cancer drugs are generally inhibitors that down-regulated the expression of genes involved in cancer pathways (Wishart et al., 2018), high expression of miRNA markers that could inhibit the expression of genes in essential canonical pathways might be the main factors that affect the prognosis of multiple cancers (Calin and Croce, 2006).

**FIGURE 7:**
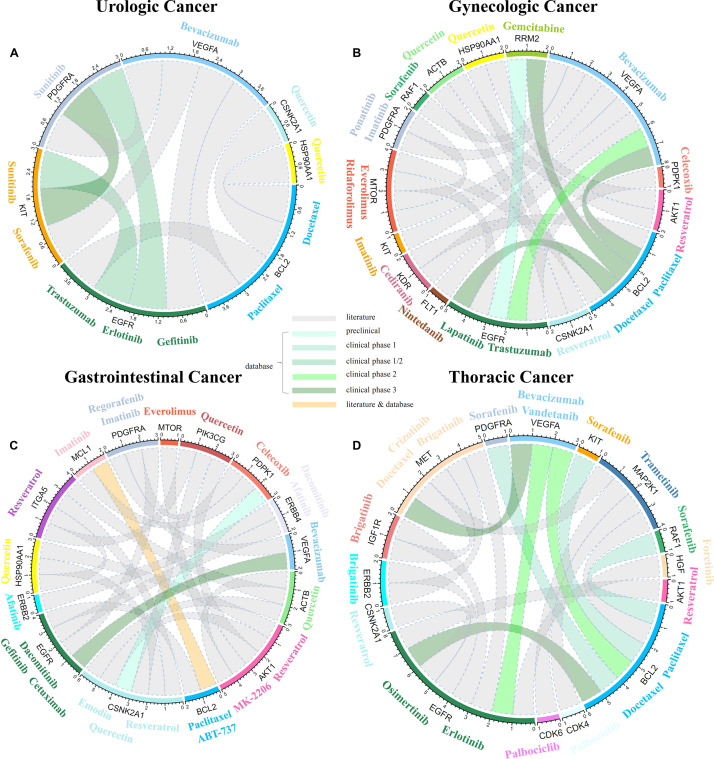
Previously detected drug combinations and corresponding drug targets. The color for each line which linked two genes represents different levels of evidence including literature, database (preclinical, clinical phase 1, clinical phase 1/2, clinical phase 2, clinical phase 3), literature and database. **(A)** Drug combinations and corresponding drug targets for urologic cancer. **(B)** Drug combinations and corresponding drug targets for gynecologic cancer. **(C)** Drug combination and corresponding drug targets for Gastrointestinal (GI) cancer. **(D)** Drug combination and corresponding drug targets for thoracic cancer.

To further reveal the potential utility of prognosis-related miRNA markers, the MOS was constructed to detect common genes in major cancer categories, which were regulated by prognosis-alternative miRNAs. As been illustrated in Figure 7, these genes have the potential of being drug targets for monotherapy or combinational therapy. For example, in urologic cancer, common genes regulated by prognosis-alternative miRNAs such as BCL2, EGFR, KIT, PDGFRA, and VEGFA, have been validated as anti-cancer drug targets previously. Also, several drug combinations that target the above gene pairs were illustrated potential application ability in cancer therapies. Pieces of evidence such as the drug combination of sunitinib targeted PDGFRA and sorafenib targeted KIT, was proved to be successful for renal cell carcinoma in clinical trial phase 3 (Liu Y. et al., 2014). Besides, the combination of erlotinib targeted EGFR and bevacizumab targeted VEGFA, was reported for the treatment of metastatic renal cell carcinoma (Hainsworth et al., 2005). In gynecologic cancers, common genes regulated by prognosis-alternative miRNAs such as BCL2, EGFR, PDGFRA, and VEGFA were also demonstrated to be combinational targets for anti-cancer drugs. Such as the combination of paclitaxel targeted BCL2 and lapatinib targeted EGFR was reported to be effective for HER2 + breast cancer in the clinical trial phase 3 (Liu Y. et al., 2014). For GI cancer, EGFR, PDGFRA, VEGFA, PDPK1, and MCL1, etc. could be validated as drug targets for anti-cancer drugs. The combination of small molecules including imatinib targeted MCL1 and celecoxib targeted PDPK1 was reported to treat the HT30 colorectal cancer (Atari-Hajipirloo et al., 2016). Beside small molecules, the combination of plant extracts including resveratrol targeted AKT1 and quercetin targeted PIK3CG were proved to be useful for colon cancer (Del Follo-Martinez et al., 2013). In thoracic cancers, common genes regulated by prognosis-alternative miRNAs such as BCL2, EGFR, IGF1R, VEGFA, and MET were validated as drug targets and the combination of docetaxel targeted MET and vandetanib targeted VEGFA was illustrated to be effective for non-small cell lung cancer in the clinical trial phase 3 (Liu Y. et al., 2014). Detailed information on potential drug combinations in literature and databases could be found in Supplementary Table 14. Among all common genes regulated by prognosis-alternative miRNAs in 19 solid tumors, BCL2, CSNK2A1, EGFR, PDGFRA, and VEGFA, etc. could be considered as drug targets for monotherapy and combinational therapy in multiple cancers. Besides, tumor multiplicity is an important clinicopathological feature, which has been proved to affect the prognosis in multiple cancers (Ajili et al., 2012; Park et al., 2018; Zhang et al., 2018). In the future, it is expected that the accumulation of available clinical information will allow us to considered the tumor multiplicity in prognosis analysis.

In this study, we comprehensively depicted the ncRNA expression profiles of 19 solid tumors from large-scale populations of the TCGA database. The contributions of this study majored in three parts: (1) identified prognosis-related ncRNA markers for 19 solid tumors and constructed the PCPA models from the perspective of ncRNAs, (2) analyzed the regulation patterns of prognostic ncRNA markers on carcinogenic pathways, (3) detected the potential drug targets for 19 solid tumors. Substantial evidence illustrated that several drug targets detected in our study were existed targets for FDA approved anti-cancer drugs. The results generated from this study could provide useful information on (1) prognosis analysis for different cancer patients, (2) target-specific drug design, which might be useful to guide clinical medication or anti-cancer drug development.

## Data Availability Statement

The lncRNA expression profiles of 19 solid tumors were obtained from The Atlas of Non-coding RNAs in Cancer (TANRIC) database (Li et al., 2015) based on the TCGA database (Tomczak et al., 2015). The miRNA expression profiles of IlluminaHiseq platform and clinical information of corresponding tumors were obtained from the TCGA module of Xena Public Data Hubs in the UCSC Xena platform (Goldman et al., 2017). And the genes which were regulated by corresponding prognosis-related miRNA markers were annotated through three high-quality experimentally validated miRNA-target interaction databases, including miRTarbase (Chou et al., 2018), miRecords (Xiao et al., 2009), and TargetScan (Riffo-Campos et al., 2016). Genes of canonical signaling pathways were downloaded from the KEGG database (Kanehisa, 2002). Anti-cancer drugs and drug combinations were derived from drugBank (Wishart et al., 2018) and DCDB (Liu Y. et al., 2014) database. The targets of anti-cancer drugs were extracted from drugBank (Wishart et al., 2018) and TTD (Li et al., 2018) database.

## Author Contributions

TQ and ZY designed the study and wrote the manuscript. ZY collected the corresponding datasets and complete *in silico* analyses. QW and XY assisted in the model construction. LZ and KT assisted in the model validation. TQ and ZC supervised the whole project and edited the manuscript.

## Conflict of Interest

The authors declare that the research was conducted in the absence of any commercial or financial relationships that could be construed as a potential conflict of interest.

## Abbreviations

AUC: area under ROC
EFA: exploratory factor analysis
KM: Kaplan–Meier
LR: logistic regression
MAPK: MAP-kinase
MOS: marker-oriented simulation
NB: Naïve Bayes
ncRNAs: non-coding RNAs
NN: neural network
OS: overall survival
PCPA: pan-cancer prognosis analysis
PI3K: PI-3-kinase
ROC: receiver operating characteristic
SVMs: support vector machines
TANRIC: The Atlas of Non-coding RNAs in Cancer
TCGA: The Cancer Genome Atlas.
