# Supplementary material for: Reveal the Regulation Patterns of Prognosis-Related miRNAs and lncRNAs Across Solid Tumors in the Cancer Genome Atlas
Source: Front Cell Dev Biol. 2020 May 25;8:368. doi: 10.3389/fcell.2020.00368 (PMC7261917; doi:10.3389/fcell.2020.00368)
Supplement: Supplementary file 1 [file Data_Sheet_1.pdf]

Supplementary Figure

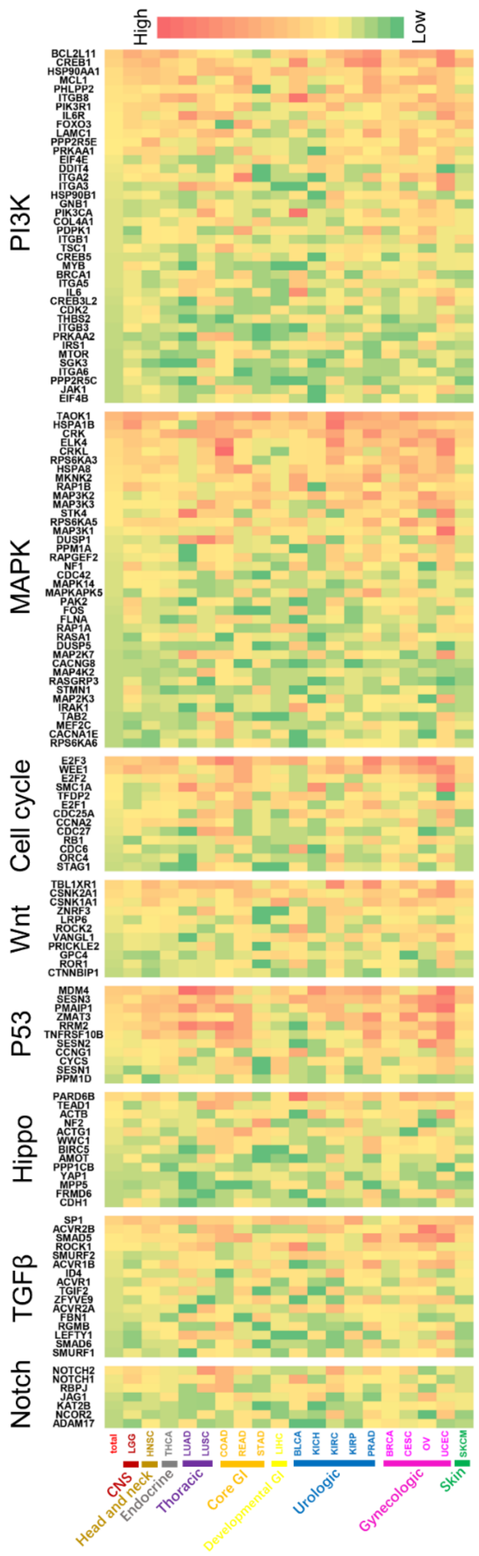

Supplementary Figure 1. The regulation patterns of genes in canonical pathways for multiple cancer types. For each pathway, the genes which were regulated by over 5% of the miRNA markers were illustrated. For each cancer type, different color represents the regulation percentage of corresponding miRNA markers.

Supplementary Table

Supplementary Table 1. The original numbers of samples and lncRNAs[a]

	miRNAs		lncRNAs	
cancer_abbreviation	*sample_count*	*feature_count*	*sample_count*	*feature_count*
LGG	524	2157	486	12727
HNSC	529	2246	468	12727
THCA	569	2217	556	12727
LUAD	495	2228	546	12727
LUSC	380	2213	237	12727
COAD	261	2113	157	12727
READ	92	2003	71	12727
STAD	428	2178	318	12727
LIHC	420	2172	250	12727
BLCA	429	2210	271	12727
KICH	89	1917	91	12727
KIRC	311	2048	515	12727
KIRP	321	2114	228	12727
PRAD	544	2111	426	12727
BRCA	832	2238	942	12727
CESC	311	2200	199	12727
OV	485	2165	412	12727
UCEC	430	2238	320	12727
SKCM	452	2220	226	12727

[a]Column 1 represents the cancer abbreviation. Column 2 to 3 represents the number of samples and miRNAs in the original miRNA expression profiles of each cancer, respectively. Column 4 to 5 represents the number of samples and lncRNAs in the original lncRNA expression profiles of each cancer, respectively.

Supplementary Table 2. The numbers of samples and ncRNAs with OS[a]

	miRNAs		lncRNAs	
cancer_abbreviation	*sample_count*	*feature_count*	*sample_count*	*feature_count*
LGG	519	287	481	12727
HNSC	483	289	424	12727
THCA	509	284	496	12727
LUAD	439	220	476	12727
LUSC	331	296	216	12727
COAD	243	245	150	12727
READ	87	283	65	12727
STAD	377	245	260	12727
LIHC	365	261	197	12727
BLCA	406	247	250	12727
KICH	65	316	65	12727
KIRC	239	247	448	12727

KIRP	284	269	195	12727
PRAD	491	254	373	12727
BRCA	751	225	824	12727
CESC	295	274	194	12727
OV	483	211	410	12727
UCEC	397	248	316	12727
SKCM	439	235	221	12727

[a]Column 1 represents the cancer abbreviation. Column 2 to 3, as well as 4 to 5, represents the number of samples and miRNAs, and the number of samples and lncRNAs in expression profiles after intersecting with samples with OS.

Supplementary Table 3. The median OS in 19 solid tumors[a]

cancer_abbrevation	***median_OS (days)***
LGG	706
HNSC	653.5
THCA	955
LUAD	699
LUSC	683.5
COAD	730.5
READ	673
STAD	437.5
LIHC	624
BLCA	523
KICH	2024
KIRC	1217
KIRP	786
PRAD	924
BRCA	931
CESC	659
OV	1021
UCEC	917
SKCM	1124

[a]Column 1 represents the cancer abbreviation. Column 2 represents the median OS evaluated with the original clinical matrix of the large-scale population in the TCGA database.

Supplementary Table 4. Detailed information of 19 solid tumors[a]

Cancer abbreviation	***TCGA name***	***category***
LGG	TCGA Lower Grade Glioma	Cancers of the central nervous system
HNSC	TCGA Head and Neck Cancer	Head and neck
THCA	TCGA Thyroid Cancer	Endocrine

LUAD	TCGA Lung Adenocarcinoma	Thoracic
LUSC	TCGA Lung Squamous Cell Carcinoma	Thoracic
COAD	TCGA Colon Cancer	Core gastrointestinal
READ	TCGA Rectal Cancer	Core gastrointestinal
STAD	TCGA Stomach Cancer	Core gastrointestinal
LIHC	TCGA Liver Cancer	Developmental gastrointestinal
BLCA	TCGA Bladder Cancer	Urologic
KICH	TCGA Kidney Chromophobe	Urologic
KIRC	TCGA Kidney Clear Cell Carcinoma	Urologic
KIRP	TCGA Kidney Papillary Cell Carcinoma	Urologic
PRAD	TCGA Prostate Cancer	Urologic
BRCA	TCGA Breast Cancer	Gynecologic
CESC	TCGA Cervical Cancer	Gynecologic
OV	TCGA Ovarian Cancer	Gynecologic
UCEC	TCGA Endometrioid Cancer	Gynecologic
SKCM	TCGA Melanoma	Melanocytic cancers of the skin

[a] Column 1 to 3 represents the abbreviation, TCGA name, and category of cancers involved in this study.

Supplementary Table 5. Prognosis-related ncRNAs[a]

[a]For each cancer, column 1 and column 2 represent the prognosis-related miRNAs and lncRNAs respectively. The results of different cancers were presented on different sheets.

Supplementary Table 6. The numbers of samples and ncRNAs in PCPA modeling[a]

	**miRNAs**			**lncRNAs**			**ncRNAs**		
cancer_abbreviation	***sample***	***train***	***feature***	***sample***	***train***	***feature***	***sample***	***train***	***feature***
LGG	519	347	73	481	322	95	488	345	168
HNSC	483	324	89	424	284	34	384	259	123
THCA	509	340	73	496	332	20	499	343	93
LUAD	439	294	26	476	318	16	409	276	42
LUSC	331	222	27	216	145	35	63	44	15
COAD	243	164	20	150	101	8	0	~	~
READ	87	59	27	65	44	5	0	~	~
STAD	377	253	25	260	174	13	233	157	38
LIHC	365	245	36	197	133	55	190	128	91
BLCA	406	272	18	250	168	35	250	168	53
KICH	65	45	26	65	45	28	65	45	31
KIRC	239	160	46	448	300	56	173	117	102
KIRP	284	191	40	195	131	18	190	128	58
PRAD	491	329	44	373	250	30	368	247	74
BRCA	751	502	90	824	551	28	505	346	118
CESC	295	198	48	194	131	48	194	133	96
OV	483	324	32	410	275	13	404	281	45
UCEC	397	266	24	316	210	1	177	122	25

SKCM	439	294	59	221	149	84	222	151	143

[a]Column 1 represents the cancer abbreviation. Column 2 to 4, 5 to 7, 8 to 10, represents the number of samples, training samples, features for miRNAs, lncRNAs and ncRNAs in PCPA modeling, respectively.

Supplementary Table 7. The AUC values of 4 methods in PCPA modeling[a]

	**miRNAs**				**lncRNAs**				**ncRNAs**			
cancer_abbrevation	***NN***	***LR***	***NB***	***SVM***	***NN***	***LR***	***NB***	***SVM***	***NN***	***LR***	***NB***	***SVM***
LGG	0.51	0.62	0.64	0.62	0.62	0.67	0.69	0.32	0.55	0.62	0.60	0.67
HNSC	0.61	0.61	0.73	0.67	0.86	0.77	0.88	0.7	0.53	0.52	0.82	0.64
THCA	0.59	0.67	0.69	0.68	0.67	0.75	0.78	0.24	0.63	0.68	0.75	0.68
LUAD	0.55	0.64	0.68	0.65	0.73	0.67	0.74	0.46	0.61	0.66	0.72	0.69
LUSC	0.51	0.67	0.68	0.67	0.75	0.8	0.87	0.22	0.69	0.44	0.73	0.49
COAD	0.46	0.51	0.63	0.56	0.72	0.85	0.86	0.81	~	~	~	~
READ	0.62	0.74	0.86	0.88	0.7	0.94	0.76	0.97	~	~	~	~
STAD	0.46	0.69	0.74	0.63	0.73	0.71	0.75	0.66	0.50	0.76	0.82	0.63
LIHC	0.28	0.67	0.78	0.72	0.71	0.68	0.88	0.62	0.58	0.73	0.83	0.61
BLCA	0.44	0.74	0.76	0.64	0.65	0.81	0.91	0.74	0.38	0.67	0.91	0.66
KICH	0.42	0.79	0.75	0.7	0.96	0.99	1	0.98	0.83	0.82	0.85	0.68
KIRC	0.73	0.73	0.74	0.78	0.65	0.64	0.65	0.66	0.68	0.75	0.61	0.81
KIRP	0.42	0.65	0.69	0.64	0.69	0.66	0.74	0.61	0.58	0.51	0.76	0.39
PRAD	0.61	0.68	0.72	0.64	0.81	0.76	0.81	0.73	0.60	0.71	0.87	0.75
BRCA	0.52	0.63	0.69	0.69	0.63	0.69	0.7	0.72	0.48	0.65	0.68	0.72
OV	0.57	0.58	0.64	0.6	0.72	0.71	0.71	0.31	0.69	0.67	0.71	0.67
CESC	0.49	0.73	0.79	0.68	0.45	0.83	0.86	0.21	0.73	0.79	0.93	0.78
SKCM	0.71	0.75	0.83	0.72	0.71	0.81	0.88	0.65	0.56	0.55	0.89	0.58
UCEC	0.74	0.67	0.75	0.68	0.47	0.53	0.53	0.53	0.60	0.59	0.69	0.66

[a]Column 1 represents the cancer abbreviation. Column 2 to 5, 6 to 9, 10 to 13, represents the AUC values of NN, NB, LR and SWM method for miRNAs, lncRNAs and ncRNAs (including miRNA and lncRNA) in PCPA modeling, respectively. For COAD and READ, there are no overlapped samples for miRNA and lncRNA expression profiles, thus the AUC of ncRNAs were marked as "~"

Supplementary Table 8. Log-rank p values of survival analysis on 19 solid tumors[a]

cancer abbreviation	*miRNAs*	*lncRNAs*	*ncRNAs*
LGG	<0.0001	<0.0001	<0.0001
HNSC	0.034	<0.0001	<0.0001
THCA	0.93	0.54	0.61
LUAD	0.038	0.00028	0.0023
LUSC	0.052	<0.0001	0.082
COAD	0.91	0.066	~
READ	0.044	0.17	~
STAD	0.021	0.045	0.0017
LIHC	<0.0001	<0.0001	<0.0001

BLCA	0.013	<0.0001	<0.0001
KICH	0.0028	0.0046	0.063
KIRC	0.24	<0.0001	0.00017
KIRP	0.16	0.00013	0.67
PRAD	0.2	0.66	1
BRCA	0.075	0.005	0.95
CESC	0.011	0.011	0.0089
OV	0.43	0.0013	0.49
UCEC	<0.0001	0.35	0.32
SKCM	0.0015	<0.0001	<0.0001

[a] Column 1 represents the abbreviation of cancers, and column 2 to 4 represents the log-rank p values of survival analysis based on prognosis-related miRNAs, lncRNAs and ncRNAs (including miRNA and lncRNA) respectively.

Supplementary Table 9. Prognosis-related ncRNA markers in 19 solid tumors[a]

[a] Each row represents the name of prognosis-related ncRNA markers and corresponding cancer types. Results of miRNA and lncRNA were presented in different sheets.

Supplementary Table 10. Refined gene-specific pathways[a]

[a] Each sheet represents the genes in one of the refined gene-specific pathways.

Supplementary Table 11. Prognosis-alternative miRNAs through MOS in 4 category cancers

Supplementary Table 12. Common genes regulated by miRNA markers in 4 major cancer categories[a]

[a] Column 1 represents the name of common genes regulated by miRNA markers in each cancer category, and remain columns represent the pathways involved by these common genes. Results of different cancer categories were presented in different sheets.

Supplementary Table 13. Potential drug targets in 4 category cancers

Supplementary Table 14. Known drug combinations in 4 category cancers

Supplementary Table 14a. Known drug combinations in urologic cancers [a]

target1	drug1	target2	drug2	database/PMID	cancer
BCL2	Docetaxel	VEGFA	bevacizumab	18276061	prostate cancer phase 2
BCL2	Docetaxel	EGFR	trastuzumab	11685722	advanced prostate cancer
BCL2	paclitaxel	CSNK2A1	Quercetin	32021294	Prostate Cancer
BCL2	paclitaxel	HSP90AA1	Quercetin	32021294	Prostate Cancer
EGFR	gefitinib	KIT	Sunitinib	DCDB	Renal cell carcinoma phase 1/2
EGFR	gefitinib	PDGFRA	Sunitinib	DCDB	Renal cell carcinoma phase 1/2
EGFR	erlotinib	VEGFA	bevacizumab	16204015	metastatic renal cell carcinoma
KIT	Sorafenib	PDGFRA	Sunitinib	DCDB	Renal cell carcinoma phase 3
KIT	Sorafenib	VEGFA	bevacizumab	19402058	advanced renal cell carcinoma

PDGFRA	Sunitinib	VEGFA	bevacizumab	19402058	advanced renal cell carcinoma

[a] Column 1 and 3 stands for the common genes regulated by prognosis-alternative miRNAs in urologic cancers, and column 2 and 4 stands for the corresponding drugs targeted genes in column 1 and 2 respectively. Columns 5 and 6 represent the evidence and indication of this drug pairs in columns 2 and 4 respectively.

Supplementary Table 14b. Known drug combinations in gynecologic cancers [a]

target1	drug1	target2	drug2	database/PMID	cancer
AKT1	Resveratrol	ACTB	Quercetin	18607509	breast cancer
AKT1	Resveratrol	HSP90AA1	Quercetin	18607509	breast cancer
BCL2	Paclitaxel	MTOR	everolimus	20975068	Metastatic Breast Cancer
BCL2	Paclitaxel	EGFR	Lapatinib	DCDB	HER2+ Breast cancer phase 3
BCL2	Docetaxel	RRM2	Gemcitabine	DCDB	metastatic Breast cancer phase 3
BCL2	Paclitaxel	VEGFA	Bevacizumab	DCDB	metastatic Breast cancer phase 3
BCL2	paclitaxel	KDR	Cediranib	26474517	metastatic or recurrent cervical cancer
CSNK2A1	Resveratrol	ACTB	Quercetin	18607509	breast cancer
CSNK2A1	Resveratrol	HSP90AA1	Quercetin	18607509	breast cancer
EGFR	Lapatinib	MTOR	everolimus	20624784	primary breast cancer
EGFR	Trastuzumab	RRM2	Gemcitabine	DCDB	Breast cancer preclinical
EGFR	Trastuzumab	VEGFA	Bevacizumab	DCDB	Breast cancer phase 2
EGFR	trastuzumab	PDPK1	celecoxib	15217939	metastatic breast cancer phase II
FLT1	nintedanib	VEGFA	Bevacizumab	24864163	advanced ovarian cancer
KDR	Cediranib	VEGFA	Bevacizumab	24864163	advanced ovarian cancer
KIT	Imatinib	VEGFA	Bevacizumab	24864163	advanced ovarian cancer
MTOR	ridaforolimus	PDGFRA	ponatinib	23468082	mutant endometrial cancer
MTOR	everolimus	VEGFA	Bevacizumab	20624784	primary breast cancer
PDGFRA	Imatinib	VEGFA	Bevacizumab	24864163	advanced ovarian cancer
RAF1	Sorafenib	VEGFA	Bevacizumab	24864163	advanced ovarian cancer

[a] Column 1 and 3 stands for the common genes regulated by prognosis-alternative miRNAs in gynecologic cancers, and column 2 and 4 stands for the corresponding drugs targeted genes in column 1 and 2 respectively. Columns 5 and 6 represent the evidence and indication of this drug pairs in columns 2 and 4 respectively.

Supplementary Table 14c. Known drug combination in GI cancer [a]

target1	drug1	target2	drug2	database/PMID	cancer
ACTB	Quercetin	CSNK2A1	Resveratrol	23530649	colon cancer
ACTB	Quercetin	ITGA5	Resveratrol	23530649	colon cancer
ACTB	Quercetin	AKT1	Resveratrol	23530649	colon cancer
AKT1	MK-2206	MTOR	Everolimus	24416349	PTEN mutant gastric cancer
AKT1	Resveratrol	PIK3CG	Quercetin	23530649	colon cancer
AKT1	Resveratrol	CSNK2A1	Quercetin	23530649	colon cancer
AKT1	Resveratrol	HSP90AA1	Quercetin	23530649	colon cancer
BCL2	paclitaxel	EGFR	gefitinib	15494644	human gastric carcinoma cells
BCL2	ABT-737	MCL1	Imatinib	DrugCombDB	gastrointestinal stromal tumor

BCL2	ABT-737	MCL1	Imatinib	21115411	gastrointestinal stromal tumor
CSNK2A1	Emodin	PDPK1	Celecoxib	DCDB	liver cancer preclinical
CSNK2A1	Resveratrol	PIK3CG	Quercetin	23530649	colon cancer
CSNK2A1	Resveratrol	HSP90AA1	Quercetin	23530649	colon cancer
CSNK2A1	Quercetin	ITGA5	Resveratrol	23530649	colon cancer
EGFR	dacomitinib	ERBB4	Afatinib	24685132	colon cancer
EGFR	cetuximab	PDGFRA	regorafenib	25838391	colorectal cancer
EGFR	Cetuximab	VEGFA	Bevacizumab	DCDB	colorectal cancer phase 3
ERBB2	Afatinib	ERBB4	Dacomitinib	24685132	colon cancer
HSP90AA1	Quercetin	ITGA5	Resveratrol	23530649	colon cancer
ITGA5	Resveratrol	PIK3CG	Quercetin	23530649	colon cancer
MCL1	Imatinib	PDPK1	Celecoxib	26950454	HT30 colorectal cancer
PDGFRA	Imatinib	PDPK1	Celecoxib	26950454	HT29 colorectal cancer
PDGFRA	Imatinib	VEGFA	Bevacizumab	23108698	advanced colorectal cancer

[a] Column 1 and 3 stands for the common genes regulated by prognosis-alternative miRNAs in GI cancers, and column 2 and 4 stands for the corresponding drugs targeted genes in column 1 and 2 respectively. Columns 5 and 6 represent the evidence and indication of this drug pairs in columns 2 and 4 respectively.

Supplementary Table 14d. Known drug combinations in thoracic cancers [a]

target1	drug1	target2	drug2	database/PMID	cancer
AKT1	Resveratrol	EGFR	Erlotinib	25895606	non-small cell lung cancer
BCL2	Docetaxel	EGFR	Vandetanib	DCDB	non-small cell lung cancer phase 3
BCL2	Paclitaxel	PDGFRA	Sorafenib	DCDB	non-small cell lung cancer phase 1
BCL2	Paclitaxel	RAF1	Sorafenib	DCDB	non-small cell lung cancer phase 1
BCL2	Paclitaxel	VEGFA	Bevacizumab	DCDB	non-small cell lung cancer phase 2
BCL2	Paclitaxel	KIT	Sorafenib	DCDB	non-small cell lung cancer phase 1
BCL2	Docetaxel	MAP2K1	Trametinib	26728409	advanced non-small cell lung cancer
CDK4	Palbociclib	MAP2K1	Trametinib	26728409	Non-Small CellLung Cancers
CDK6	Palbociclib	MAP2K1	Trametinib	26728409	Non-Small CellLung Cancers
CSNK2A1	Resveratrol	EGFR	Erlotinib	25895606	non-small cell lung cancer
EGFR	osimertinib	ERBB2	Brigatinib	28287083	non-small-cell lung cancer
EGFR	osimertinib	IGF1R	Brigatinib	28287083	non-small-cell lung cancer
EGFR	osimertinib	MET	Brigatinib	28287083	non-small-cell lung cancer
EGFR	Erlotinib	VEGFA	Bevacizumab	DCDB	non-small cell lung cancer phase 2
EGFR	Erlotinib	HGF	Foretinib	29050231	advanced non-small cell lung cancer
ERBB2	Brigatinib	MET	crizotinib	29075144	non-small cell lung cancer
IGF1R	Brigatinib	MET	crizotinib	29075144	non-small cell lung cancer
MET	Docetaxel	MAP2K1	Trametinib	26728409	advanced non-small cell lung cancer
MET	Docetaxel	VEGFA	Vandetanib	DCDB	non-small cell lung cancer phase 3

[a] Column 1 and 3 stands for the common genes regulated by prognosis-alternative miRNAs in thoracic cancers, and column 2 and 4 stands for the corresponding drugs targeted genes in column 1 and 2 respectively. Columns 5 and 6 represent the evidence and indication of this drug pairs in columns 2 and 4 respectively.
